# A redescription of *Rhysida celeris* (Humbert & Saussure, 1870), with a proposal of eight new synonyms (Scolopendromorpha, Scolopendridae, Otostigminae)

**DOI:** 10.3897/zookeys.258.4675

**Published:** 2013-01-15

**Authors:** Amazonas Chagas-Júnior

**Affiliations:** 1 Laboratório Especial de Coleções Zoológicas, Instituto Butantan. Avenida Vital Brasil, 1500, 05503-900, São Paulo, SP, Brasil

**Keywords:** Neotropic, Venezuela, Taxonomy, Chilopoda, *Rhysida*

## Abstract

Seven species of the genus *Rhysida* Wood, 1862 from Venezuela and one subspecies from Peru described by Manuel Angel González Sponga and Wolfgang Bücherl respectively, are revised. *Rhysida caripensis* González-Sponga, 2002, *Rhysida neoespartana* González-Sponga, 2002, *Rhysida guayanica* González-Sponga, 2002, *Rhysida maritima* González-Sponga, 2002, *Rhysida monaguensis* González-Sponga, 2002, *Rhysida porlamarensis* González-Sponga 2002, *Rhysida sucupanensis* González-Sponga, 2002 and *Rhysida celeris andina* Bücherl, 1953 are junior synonyms of *Rhysida celeris* (Humbert & Saussure, 1870), which is redescribed and illustrated for the first time. Its geographic distribution is updated and a map showing its distribution is presented.

## Introduction

*Rhysida* is the second largest genus of the Otostigminae with circa 40 species in the Neotropics, the Indo-Malayan region and west, central and east Africa (Bonato et al. 2011). Its phylogenetic position within the tribe Otostigminae is uncertain, but Koch (1983, 1985) pointed out that *Rhysida* is closer to *Ethmostigmus* Pocock, 1898 than *Otostigmus* Porat, 1876. The Australian species of *Rhysida* were revised by Koch (1985), but the remaining species of the genus still require study. In the Neotropics, *Rhysida* is represented by 14 species and two subspecies (Minelli et al. 2006) distributed throughout Central and South America and the Greater and Lesser Antilles. Twelve species and the two subspecies are known only from the Neotropical region, however *Rhysida immarginata* (Porat, 1876) and *Rhysida longipes* (Newport, 1845) are widely distributed and can also be found in the Indo-Malayan and African regions. The species *Rhysida nuda* (Newport, 1845) has been reported as occurring in the Neotropics by several authors, namely Kraepelin (1903), Attems (1930) and Bücherl (1939, 1942, 1974). It has also been recorded from Indo-Australian region and West Africa (Attems 1930), but Koch (1983) stated that this species is exclusive to Australia and that the name *Rhysida immarginata* (Porat, 1876) appears applicable to some or even most of the overseas forms previously recorded as *Rhysida nuda*.

In a paper on myriapods from Venezuela, González-Sponga (2002) described seven new species of *Rhysida*, and redescribed *Rhysida longipes* based on four specimens from an urban area of Caracas. As the descriptions of the seven new species are brief and the illustration presented by González-Sponga (2002) didn’t explicitly show the diagnostic features that separate the new species from *Rhysida celeris*, known to Venezuela, their type specimens have been herein revised. *Rhysida celeris andina* from Peru is also revised. As no illustrations are known for *Rhysida celeris* and its geographical variation is unknown, I redescribe and illustrate it for the first time. The redescription of *Rhysida celeris* is based in 44 specimens from several localities of Antilles and Central and South Americas. This work is the first of a series that are in progress on species of Scolopendromorpha from Venezuela described by Manuel Ángel González Sponga. Repository acronyms are as follows: BMNH—Natural History Museum, London, UK; ICN—Intituto de Ciencias Naturales de La Universidad Nacional de Colombia, Bogotá, Colombia; IAVH—Colección Zoológica – Instituto de Investigación de Recursos Biológicos – Alexander Von Humboldt, Villa de Leyva, Colombia; MIZA—Museo del Intituto de Zoología Agrícola Francisco Fernández Yèpez, Maracay, Venezuela; NMNH—National Museum of Natural History, Smithsonian Institution, Washington, D. C., USA; QCAZ—Museo de Zoología de La Pontificia Universidad Católica del Ecuador, Quito, Ecuador; ZMH—Zoologisches Museum Hamburg, Hamburg, Germany; ZMUC—Natural History Museum of Denmark (Zoologisk Museum), Copenhagen, Denmark. Descriptive terminology follows Bonato et al. (2010).

## Taxonomy

### Rhysida celeris

(Humbert & Saussure, 1870)

http://species-id.net/wiki/Rhysida_celeris

**Figure 1–4.**
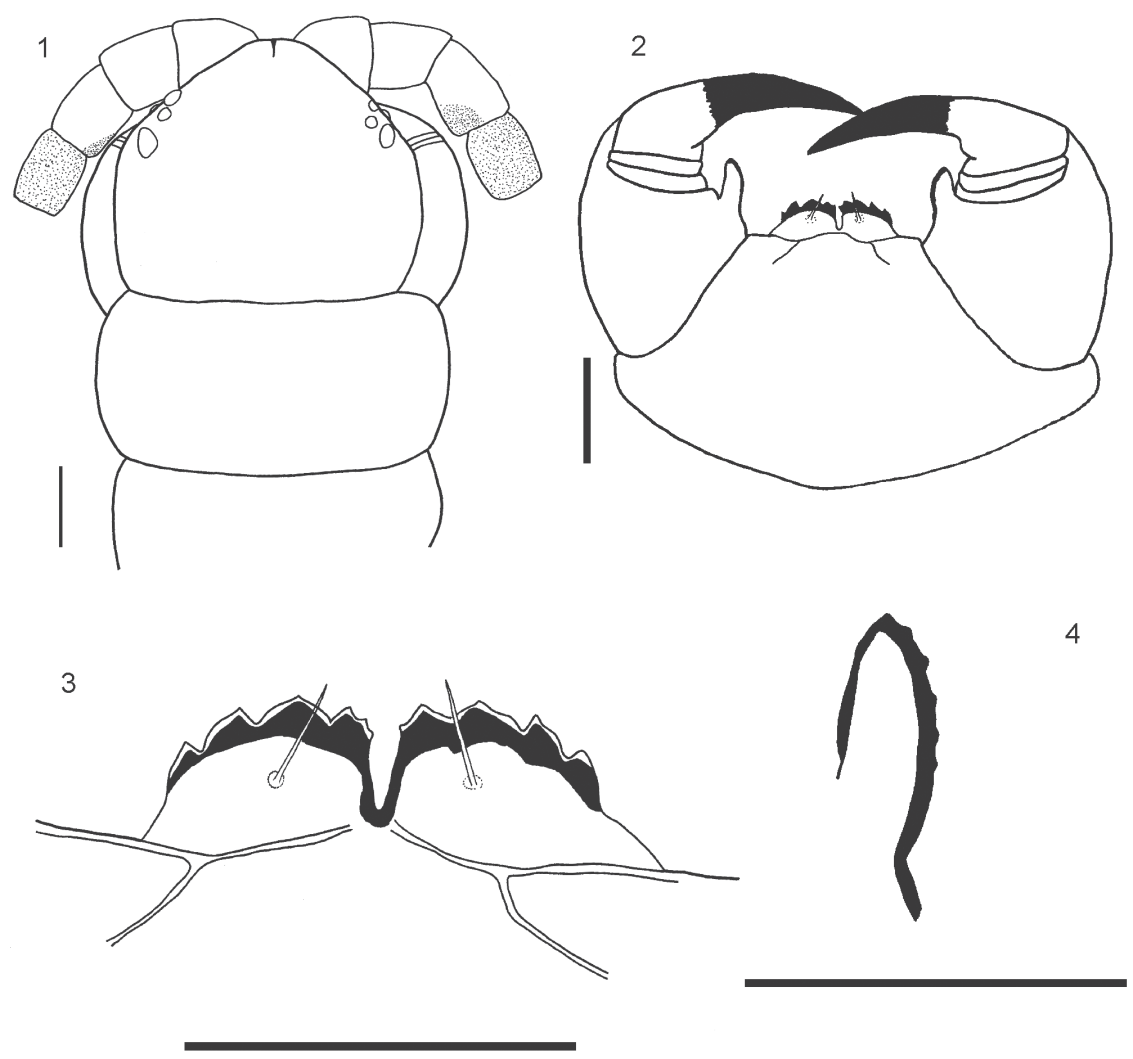
**1** *Rhysida celeris* from Ecuador. Cephalic plate **2** Forcipular Coxosternum **3** Tooth plates **4** Forcipular trochanteroprefemur process. Scale bars 1 mm.

Figures 1 [Fig F2] –10

Branchiostoma celer Humbert & Saussure, 1870: 202; Saussure and Humbert 1872: 122; Kohlrausch 1881: 69; Meinert 1886: 183.
Rhysida celeris Pocock, 1896: 27; Kraepelin 1903: 149 (+*Rhysida aspera*); Attems 1930: 188; Bücherl 1942: 315; 1974: 119.
Trematoptychus celeris Chamberlin, 1914: 181.
Rhysida celeris andina Bücherl, 1953: 112; 1974: 119. Syn. n.
Rhysida caripensis González-Sponga, 2002: 51. Syn. n.
Rhysida neoespartana González-Sponga, 2002: 52. Syn. n.
Rhysida guayanica González-Sponga, 2002: 53. Syn. n.
Rhysida maritima González-Sponga, 2002: 54. Syn. n.
Rhysida monaguensis González-Sponga, 2002: 55. Syn. n.
Rhysida porlamarensis González-Sponga, 2002: 56. Syn. n.
Rhysida sucupanensis González-Sponga, 2002: 58. Syn. n.

#### Type material examined.

**Venezuela** –***Rhysida caripensis*** – Holotype (MIZA0015958, ex. MAGS 680a) and paratype (MIZA0015958, ex. MAGS 680b), Edo. Monagas, Caripe, Caripe (10°12'00"N, 63°30'00"W), ix.1991, Dora Padrón de García leg.; ***Rhysida neoespartana*** – Holotype (MIZA0015954, ex. MAGS 319a) and paratype (MIZA0015954, ex. MAGS 319b), Edo. Nueva Esparta, La Asunción, Marcano (11°02'00"N, 63°51'46"W), 1988–1989–1991, Dora Padrón de Garcia leg.; ***Rhysida guayanica*** – Holotype (MIZA0015961, ex. MAGS 738a) and paratype (MIZA0015961, ex. MAGS 738b), Edo. Bolívar, Los Pijiguaos, Cedeño (06°35'20"N, 66°45'12"W), 01.v.1992, José Manuel Ayala leg.; ***Rhysida maritima*** – Holotype (MIZA0015957, ex. MAGS 580a), Edo. Carabobo, Goaigoaza, Puerto Cabello (10°29'36"N, 68°02'48"W) in 19.iii.1990, and paratype (MIZA0015963, ex. MAGS 1002), Edo. Carabobo, San Esteban, Puerto Cabello, in 18.x.1998, both collected by A. R. Delgado de González & Manuel Ángel González Sponga; ***Rhysida monaguensis*** – Holotype (MIZA0016084, ex. MAGS 970a) and paratype (MIZA0016084, ex. MAGS 970b), Edo. Monagas, Uverito, Sotillo (08°42'10"N, 63°22'00"W), 30.iv.1998, Oswaldo Fuentes leg.; ***Rhysida porlamarensis*** – Holotype (MIZA0015953, ex. MAGS 315a and paratype MIZA0015953, ex. MAGS 315b), Edo. Nueva Esparta, Porlamar, Calle San Rafael, Porlamar (10°57'30"N, 63°51'00"W), 30.iii.1988, Carlos Contreras leg.; ***Rhysida sucupanensis*** – Holotype (MIZA0015908, ex. MAGS 63a) and paratype (MIZA0015908, ex. MAGS 63b), Edo. Delta Amaruco, Sucupana, Casacoima (08°41'N, 61°48'W), 16.ix.1987, Pedro Delgado leg.

#### Additional material examined.

**Jamaica** – BMNH – 1 specimen; **Dominican Republic** – ZMH – 4 specimens, Sánchez, 5.vii.1905, C. Gagzol; ZMH – 1 specimen, Sánchez, Bay V. Samaná, xii.1894, Bock; **Haiti** – ZMH – 4 specimens, Port au Prince, 29.v.1901, Dr. Rouch; **Montserrat** – BMNH – 1 specimen, B.W.I., ii.1932; **Costa Rica** – ZMH – 1 specimen, Puerto Simón, 19.xi.1899, R. Mull; **Suriname** – ZMH – 2 specimens, Paramaribo, 28.v.1910, C. Hellesl; **Colombia** – IAVH – 1 specimen, Vichada, Cumaribo, Cgto. Santa Rita, PNN El Tuparro, 14-16-II-2004, I. Quintero, E. Gonzalez; IAVH – 1 specimen, Vichada, Cumaribo, Selva de Mataven, 31-III-02-IV-2007, L.E. Franco; IAVH – 1 specimen, Vichada, Gaviotas, 31-VI-1995; IAVH – 1 specimen, Caquetá, Solano, PNN Chiribiquete, 24-26-II- 2000, M. Ospina & E. González; ICN-M.Ch-0005, 1 specimen, Amazonas, Leticia, Via Torame, X-2000, Sist. Animal; ICN-M.Ch-0035, 1 specimen, Sucre, San Onofre, Boca cerrada, canal del dique, 1-IV-2000, E. Ulloa; ICN-M.Ch-0037, 1 specimen, Sucre, Galeras, Vereda corozera, 14-I-1999, E. Hernández; ICN-M.Ch-0039, 1 specimen, Meta, Vereda Apiay, sector el bosque villa Lolé , 23-XII-2001, M. Rojas; ICN-M.Ch-0097, 1 specimen, Casanare, Aguazul, Vda. El Charte, Finca Namaste, 20-IX-1996, Estudiantes Biol UN; ICN-M.Ch-0100, 1 specimen, Tolima, San Luis, 13-VI-1992, A. Castillo; ICN-M.Ch-0136, 1 specimen, Meta, San Martin, 1–15 -IV-2011, W. Galvis; ICN-M.Ch-0138, 1 specimen, Vaupés, Est. Biol. Caparù, 2002–2004, J. Pinzón; **Ecuador** – QCAZ – 3 specimens, Francisco Orelanna, La Joya de Los Sachas, 3.vi.2006, J. Mideros; QCAZ – 1 specimen, Francisco Orelanna, PN Yasumi, M.J. Tamariz; QCAZ – 1 specimen, Napo, Tena, 19.ix.2004; **Argentina** – ZMUC – 11 specimens, Riacho del Oro, 17.06.1899, W. Sorensen; ZMUC – 2 specimens, 19.08.1895.

#### Diagnosis.

General body color light blue or olive green, sternites and legs light blue or yellowish; prefemur and femur of the ultimate legs light blue, sometimes tibiae and tarsi are pale. Antennae with 17 to 21 articles, first two articles, dorsal surface and ¾ of ventral surface of third articles glabrous. Cephalic plate smooth, without sutures or depressions; tooth plates wider than high, 4+4, 4+5 or 5+5 teeth. Tergites smooth; complete paramedian sutures present from tergites 3–5 to 19–20, margination from tergites 5–9 to 21. Posterior border of tergite 21 ending in an obtuse angle. A pair of spiracles at 7th leg -bearing segment. Sternites 2, 3 or 4 to 19 with anterior incomplete paramedian sutures, but without depressions. Coxopleuron not prolonged, very short process with two small apical spines. Legs 1 to 16 (or 17) or 2 to 18 (sometimes 17) with two tarsal spurs, 19 (or 20) with one and 21 without; ultimate legs long, prefemur without spines.

#### Redescription.

**Figure 5–8. F2:**
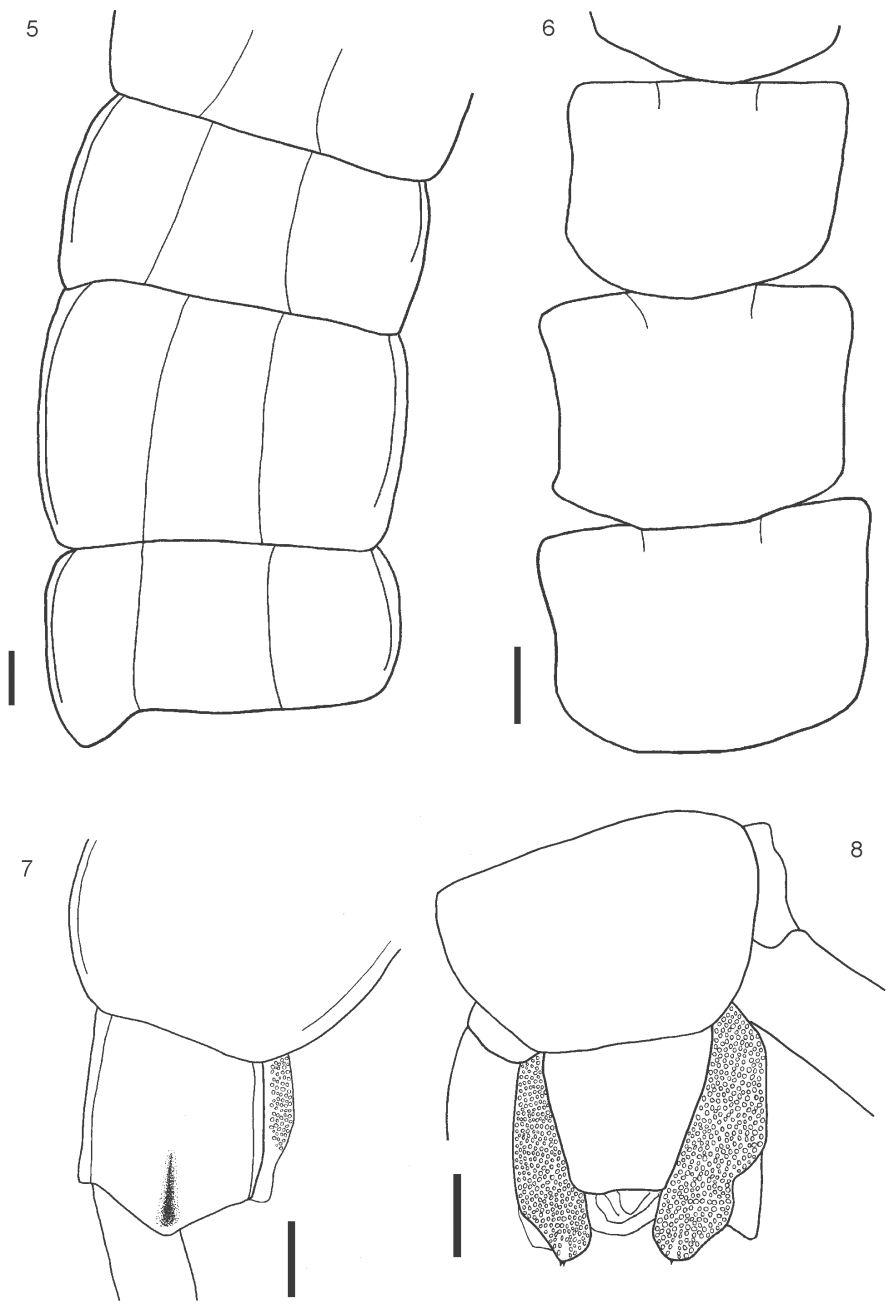
**5** Tergites 11, 12 and 13 **6** Sternites 4, 5 and 6 **7** Tergite 21 **8** Segment 21 showing sternite 21 and coxopleuron. Scale bars 1 mm.

**Figure 9–10.**
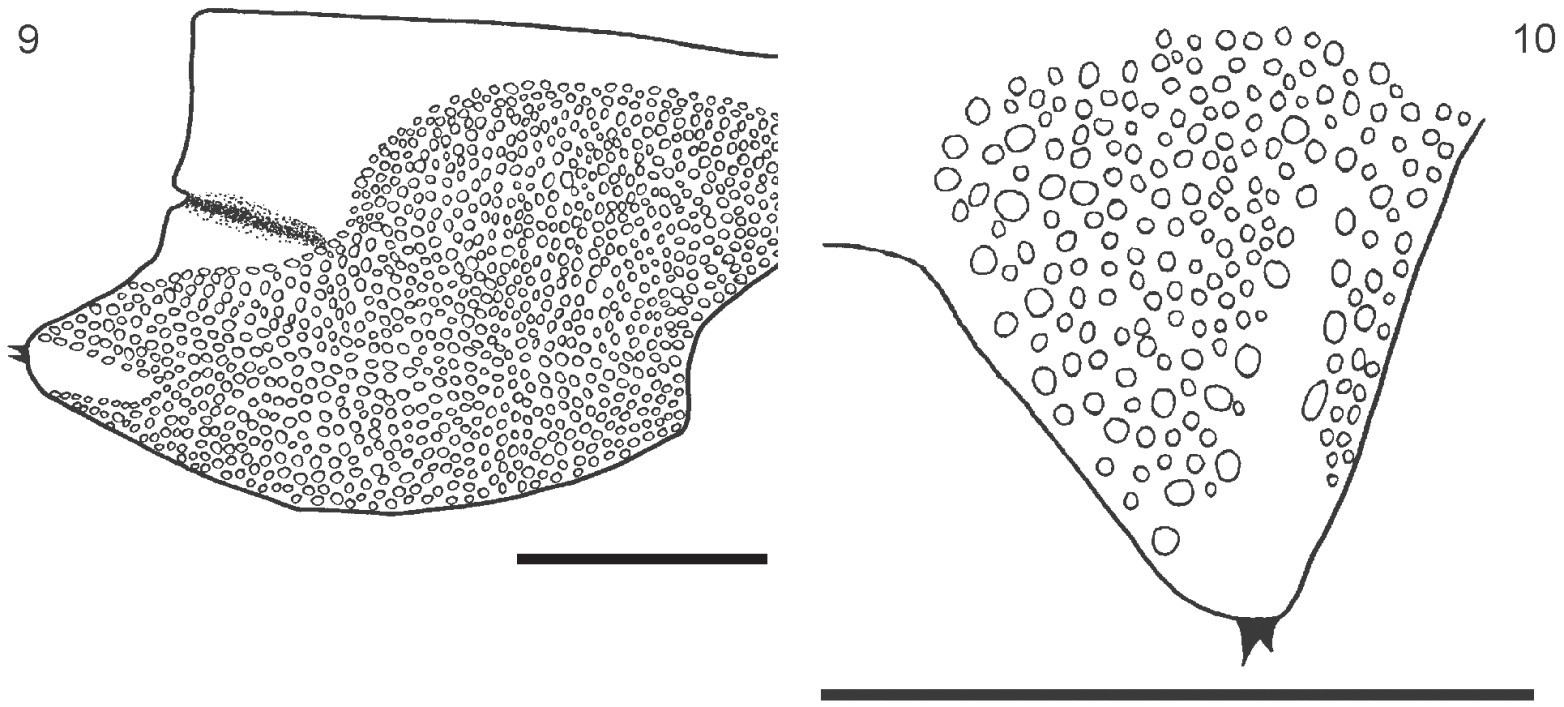
**9** Segment 21 showing the coxopleuron **10** Detail of the terminal part of the coxopleuron showing the spines. Scale bar 1 mm.

Body length from 40 to 70 mm. General body color light blue or olive green, sternites and legs light blue or yellowish; prefemur and femur of the ultimate legs light or dark blue, but sometimes tibia and tarsi are pale or light blue. Antennae with 17 to 21 articles, first two and dorsal surface and ¾ of the ventral surface of the third article are glabrous; antennae reaching the posterior margin of tergite 5. Cephalic plate smooth, with a median sulcus but without sutures or depressions (Fig. 1), wider than long. Anterior margin of cephalic plate right-angled, with four ocelli in each side, posterior margin slightly rounded (Fig. 1). Forcipular coxosternum without depressions (Fig. 2); tooth plates with 4+4, 4+5 or 5+5 teeth; the inner two teeth are closer to each other than to the external teeth (Fig. 3). Each tooth plate with a long seta in the center; the tooth plates with obtuse angled basal suture. Trochanteropreforal process well-developed, long and ending as a point, with three to five denticles laterally, these sometimes not visible (Fig. 4). Tergites smooth, wider than long. Tergite 1 without sutures, its anterior border overlapping the posterior border of the cephalic plate. Tergites 3–5 to 19–20 with complete paramedian sutures (Fig. 5). Tergites 5 to 21 marginate, usually from tergite 11 (Fig. 5), but sometimes also from tergite 16. The margination is clearly visible in the anterior part of the tergites; tergite 21 with a slight posterior depression and triangular posterior margin (Fig. 7). A pair of spiracles on the 7th leg -bearing segment. Sternites smooth, wider than long. Sternites 2 (4)-19 with short incomplete anterior sutures (Fig. 6). However, sometimes, they are overlapped by the posterior margin of the previous sternite and visible only at sternites 5–18. Sternite 21 longer than wide; converging caudad and with straight or slightly concave posterior margin (Fig. 8). Coxopleural pores of several sizes, and numerous; the pore-field occupies nearly all surface of coxopleuron except for its dorsal and posterior parts, which are free of pores, posterior part of coxopleuron with a longitudinal depression (Fig. 9); very short coxopleural process with two apical spines (Fig. 10); apical spines may be combined as 1+2 or 2+3. Leg 1 with one femoral spur and legs 1 and 2 with one tibial spurs; legs 1 to 16, 17 or from 2 to 18 with two tarsal spurs, 19 and 20 with one and 21 without. Sometimes legs 20 lack spurs. Pretarsi of all legs with two accessory spines. Ultimate legs long and slender (12 mm to 22 mm). Prefemur without spines.

#### Taxonomic remarks.

Except for *Rhysida maritima* where the holotype and paratype were separated and given different numbers (e.g., holotype, MAGS 580a, and, paratype, MAGS 1002), the type specimens of all other species described by González-Sponga (2002) were left united and were labeled with identical numbers (e.g., holotype, MAGS7400, and a paratype, MAGS740b). The acronym MAGS in the collection numbers stands for ‘Manuel Ángel Gonzalez Sponga’ and indicates that the specimens belong to his private collection, which after his death was transferred to the myriapodological collection of Museo del Intituto de Zoología Agrícola Francisco Fernández Yèpez, Maracay, Venezuela. The type series of *Rhysida caripensis*, *Rhysida neoespartana*, *Rhysida monaguensis* and *Rhysida porlamarensis* consists of more than two specimens. The recognition of the holotype is only possible by measuring the total body length, cephalic plate and ultimate legs and by checking them against the original publication (González-Sponga 2002).

The results of the review of the type-material described by González-Sponga (2002) are summarized in Table 1. The type of *Rhysida celeris andina* was not examined, but all characters (number of antennal articles, complete paramedian sutures and the margination of the tergites, sternite sutures, forcipular tooth-plates and tarsal spurs) fall within the intraspecific variation found in *Rhysida celeris*. Therefore, *Rhysida celeris andina* was also included in this revision, but the proposed synonymy is based solely on critical analysis of the published information (Bücherl 1953).

**Table 1.** Characters of the species considered in this study. Information based on personal examination of types and original data (in parentheses) as published by González-Sponga (2002) and Bücherl (1939, 1953). ? = not described.

	*Rhysida caripensis*	*Rhysida neoespartana*	*Rhysida guayanica*	*Rhysida maritima*	*Rhysida monaguensis*	*Rhysida porlamarensis*	*Rhysida socupanensis*	*Rhysida celeris andina*
Numbers of antenna articles	19, (20) and 21	(17), 19, 20, and 19+20	18, 19, 20 and (21)	19, (20-21) and , 21	18, (19), 20 and 21	19, 20, 19+20 (17)	20, (Not described)	(20)
Complete paramedian sutures on tergites	3 to 20 (3 to 20)	3 to 20 (2-3 to 20)	3 to 20 (3 to 20)	4 to 20 (4 to 20)	7 to 20 (7 to 20)	5 to 20 (5 to 20)	3 to 20 (3 to 20)	(6 to ?)
Marginate tergites	13 to 21 (16 to 21)	16 to 21 (Posterior tergites)	14 to 21 (Posterior tergites)	16 to 21 (Posterior tergites)	17 to 21 (Posterior tergites)	18 to 21 (Posterior tergites)	15 to 21 (16 to 21)	(16-19 to 21)
Incomplete sternite paramedian sutures	2 to 18 (Without)	1 to 18 (Without)	2 to 18 (Without)	4 to 17 (Without)	3 to 17 (Without)	3 to 18 (Without)	2 to 18 (Without)	(1 to 20)
Tooth plates	4+5 (5+5)	(4+4) 5+5	4+4, 4+5 and 5+5 (Not described)	4+4 (4+4)	(3+3), 4+4, 4+5 and 5+5	4+4, 4+5 and 5+5 (Not described)	5+5 (5+5)	(0+5 (damage))
2 spurs on tarsus 1 of legs	1 to 17 and 18 (Not described)	1 to 16, 17 and 18 (Not described)	2 to 17 (Described only to leg 2)	1 to 18 (Not described)	1 to 18 (On all legs)	1 to 18 (On all legs)	1 to 18 (Not described)	(1 to 19)
1 spur on tarsus 1 of legs	18, 19 and 20 (Not described)	17, 18, 19 and 20 (Not described)	1, 19 and 20 (Described only to leg 1)	19 and 20 (Not described)	19 and 20 (Not described)	19 and 20 (Not described)	19 and 20 (Not described)	(20)
No tarsal spurs on legs	21 (21)	21 (21)	21 (21)	21 (21)	21 (21)	21 (21)	21 (21)	(21)
Terminal spines on coxopleura	2 (1)	2 (2)	2 (2)	2 (2)	1+2, (2) and 2+3	1+2 (2)	2 (2)	(1)
Distribution	Venezuela	Venezuela	Venezuela	Venezuela	Venezuela	Venezuela	Venezuela	Peru

His descriptions are based only on the holotypes, and he did not describe the variation of some characters, such as the number of antennal articles, the distribution of paramedian sutures and the margination of tergites and the number of teeth of forcipular tooth-plates. For example, complete paramedian sutures are present in all of the González-Sponga’s (2002) species, however, the distribution of sutures vary. The complete paramedian sutures on tergite 3 are not as evident as on tergite 4, but the sutures always end at tergite 20. The number of teeth in forcipular tooth-plates also vary from 3+3 to 5+5.

Two other important characters that were omitted or were poorly described by González-Sponga (2002) are the incomplete paramedian sutures on the sternites and the distribution of tarsal spurs on the legs. The incomplete paramedian sutures were not mentioned in his paper, although they are clearly visible from sternites 1 to 18, being more evident on sternites 6-15. The tarsal spurs were only described for *Rhysida guayanica*, *Rhysida monaguensis* and *Rhysida porlamarensis*, but their distribution were not indicated.

*Rhysida celeris* was originally described from ‘Carolina’ (Humbert and Saussure 1870, Saussure and Humbert 1872, Underwood 1887) and later on recorded also from Georgia, USA (Pocock 1893, Kraepelin 1903, Attems 1930). Crabill (1960) stated that the North American records were not corroborated, and Shelley (2002) deleted *Rhysida celeris* from the list of Scolopendromorpha of United States. Carolina is a common toponym in several countries in the Neotropical region. It exists in Puerto Rico, Cuba, Colombia (in the departments of Cesar, Antioquia and Magdalena), in Ecuador (in the departments of Guayas and Imbabura) and in Brazil. There is also a state of Carolina in the U.S. Virgin Islands, and also “La Carolina” in both, Mexico and Argentina. I did not examine the holotype of *Rhysida celeris*, but it is hosted (in alcohol) at the Myriapodological collection of Museum of Natural History of Genève, Switzerland. The specimen is labeled “*Branchiostoma celer* Humbert & Saussure, type, from Caroline”. A second label reads “revision R. E. Crabill, back 1970” (Peter Schwendinger, personal communication). I do not know any other additional information on the type locality, therefore, the exact whereabouts of the type locality remains uncertain. However, it is more likely that the specimen had been collected somewhere in Antilles, Central or South America rather than in the USA.

#### Distribution.

Jamaica, Dominican Republic, Haiti, Nicaragua (Meinert 1886), Venezuela (Bücherl 1942, 1959), Mexico (Kraepelin 1903, Attems 1930, Bücherl 1942), Bolivia (Silvestri 1897), Argentina (Bücherl 1942), Brazil (Kraepelin 1903, Attems 1930, Bücherl 1942, Schileyko 2002), Colombia (Chamberlin 1921), Ecuador, Peru (Bücherl 1950, Kraus 1957), Suriname and Paraguay (Silvestri 1895).

## Discussion

After reviewing the type material of the genus *Rhysida* described by González-Sponga (2002), it was verified that *Rhysida caripensis*, *Rhysida neoespartana*, *Rhysida guayanica*, *Rhysida maritima*, *Rhysida monaguensis*, *Rhysida porlamarensis* and *Rhysida sucupanensis* are junior synonyms of *Rhysida celeris*. *Rhysida celeris andina* described by Bücherl (1953) is also considered to be junior synonym of *Rhysida celeris*.

**Figure 11.**
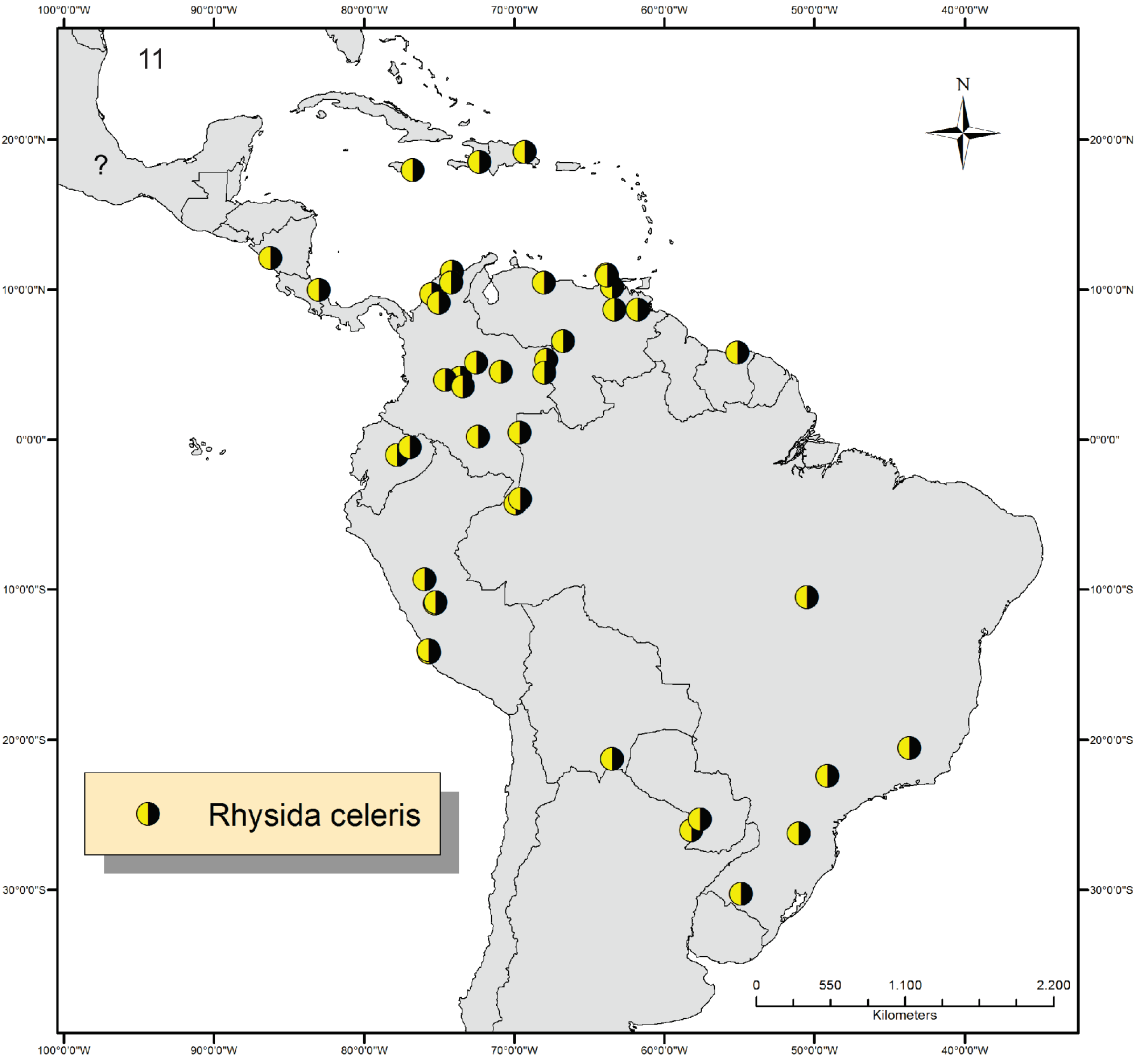
Map showing the distribution of *Rhysida celeris* in the Neotropical Region. Question mark indicates unspecified locality in Mexico.

All taxa analyzed in this study are conspecific with *Rhysida celeris* which is redescribed and illustrated based on specimens from the Antilles, Central and South America. The characters used to determine *Rhysida celeris* and their variation are summarized in Table 2. *Rhysida celeris* is the most widespread species of the genus in the Neotropical Region and is found from the sea level up to1250 meters in the mountains. The species is herewith recorded from the Dominican Republic, Haiti, Montserrat, Suriname and Ecuador for the first time (Fig. 11). Morphologically, *Rhysida celeris* is close to *Rhysida brasiliensis* Kraepelin, 1903, but differs from the latter by having complete paramedian sutures on segments 3-19/20 (vs. incomplete and short ones on segments 4-19 in *Rhysida brasiliensis*;) shorter ultimate legs (18–22 mm vs. 20–32 mm in *Rhysida brasiliensis*). With present paper, the valid species of *Rhysida* in the Neotropical Region were reduced to seven, of which five indigenous (*Rhysida celeris*, *Rhysida brasiliensis*, *Rhysida rubra* Bücherl, 1939, *Rhysida riograndensis* Bucherl, 1939 and *Rhysida chacona* Verhoeff, 1944) and two introduced (*Rhysida immarginata* and *Rhysida longipes*). Both, *Rhysida riograndensis*, from southern Brazil, and *Rhysida chacona*, from Paraguay, resemble in many aspects *Rhysida celeris*, but the lateral tergal margins are absent in both species. As the presence of lateral margins is considered an important character in Neotropical *Rhysida* and since I have not seen yet the type-material of these two species, I prefer to keep them as a valid species.

**Table 2.** Characters of *Rhysida celeris* showing the geographical variation.

	*Rhysida celeris*	*Rhysida celeris*	*Rhysida celeris*	*Rhysida celeris*
Numbers of antennal articles	17 to 21	18 to 21	17 to 21	19 to 20
Complete paramedian sutures on tergites	3 to 20	4 to 19	4 (5) to 19	3, 4 or 5 to 20
Marginates tergites	13 (14) to 21	5 to 21	10, 11, 12, 13 to 21	16 to 21
Incomplete paramedian sutures on sternites	3 (4) to 18 (19)	4 to 19	4 to 19	2, 3 or 4 to 19
Coxosternal Teeth	4+4, 4+5 and 5+5	4+4, 4+5 and 5+5	4+4, 4+5 and 5+5	4+4, 4+5 and 5+5
2 spurs on tarsus 1 of legs	1 to 16 (17 or 18)	1 to 19 or 2 to 20	2 to 18	1 to 18
1 spur on tarsus 1 of legs	19 and 20	1, 19 and 20	19 and 20	19
No tarsal spurs on legs	21	20 and 21	21	20 and 21
Terminal spines on coxopleura	1, 2 or 3	2	2	2
Specimens studied	Venezuela	Colombia	Ecuador	Costa Rica, Jamaica, Dominican Republic, Haiti, Montserrat, Suriname, and Argentina

## Supplementary Material

XML Treatment for Rhysida celeris
